# Whole body cardiovascular magnetic resonance imaging to stratify symptomatic and asymptomatic atherosclerotic burden in patients with isolated cardiovascular disease

**DOI:** 10.1186/s12880-016-0121-4

**Published:** 2016-02-29

**Authors:** Jonathan R. Weir-McCall, Suzanne L. Duce, Stephen J. Gandy, Shona Z. Matthew, Patricia Martin, Deirdre B. Cassidy, Lynne McCormick, Jill J. F. Belch, Allan D. Struthers, Helen M. Colhoun, J. Graeme Houston

**Affiliations:** 1 Division of Cardiovascular and Diabetes Medicine, Medical Research Institute, University of Dundee, ᅟ, DD1 9SY UK; 2 NHS Tayside Clinical Radiology, Ninewells Hospital, Dundee, DD1 9SY UK; 3 NHS Tayside Medical Physics, Ninewells Hospital, Dundee, DD1 9SY UK; 4 Division of Population Health Sciences, Medical Research Institute, The Mackenzie Building, University of Dundee, ᅟ, DD2 4BF UK; 5 Division of Cardiovascular and Diabetic Medicine, Level 7, Ninewells Hospital, Dundee, DD1 9SY UK

**Keywords:** Whole body magnetic resonance angiography, Atherosclerosis, Coronary artery disease, Peripheral arterial disease, Cerebrovascular disease, Atheroma burden

## Abstract

**Background:**

The aim of this study was to use whole body cardiovascular magnetic resonance imaging (WB CVMR) to assess the heart and arterial network in a single examination, so as to describe the burden of atherosclerosis and subclinical disease in participants with symptomatic single site vascular disease.

**Methods:**

64 patients with a history of symptomatic single site vascular disease (38 coronary artery disease (CAD), 9 cerebrovascular disease, 17 peripheral arterial disease (PAD)) underwent whole body angiogram and cardiac MR in a 3 T scanner. The arterial tree was subdivided into 31 segments and each scored according to the degree of stenosis. From this a standardised atheroma score (SAS) was calculated. Cine and late gadolinium enhancement images of the left ventricle were obtained.

**Results:**

Asymptomatic atherosclerotic disease with greater than 50 % stenosis in arteries other than that responsible for their presenting complain was detected in 37 % of CAD, 33 % of cerebrovascular and 47 % of PAD patients. Unrecognised myocardial infarcts were observed in 29 % of PAD patients. SAS was significantly higher in PAD patients 24 (17.5-30.5) compared to CAD 4 (2–11.25) or cerebrovascular disease patients 6 (2-10) (ANCOVA p < 0.001). Standardised atheroma score positively correlated with age (β 0.36 p = 0.002), smoking status (β 0.34 p = 0.002), and LV mass (β -0.61 p = 0.001) on multiple linear regression.

**Conclusion:**

WB CVMR is an effective method for the stratification of cardiovascular disease. The high prevalence of asymptomatic arterial disease, and silent myocardial infarctions, particularly in the peripheral arterial disease group, demonstrates the importance of a systematic approach to the assessment of cardiovascular disease.

## Background

Atherosclerosis with subsequent plaque formation is the underlying pathophysiological process in the leading causes of morbidity and mortality in the western world [1]. The widely distributed nature of atherosclerosis across the body has been appreciated for decades [2], however routine stratification or quantification of whole body disease burden is not routinely performed. Multimodal imaging studies of patients presenting with coronary artery disease [3, 4], cerebrovascular disease [5, 6] and peripheral arterial disease [7–9], have shown a high prevalence of atherosclerotic disease in other sites in addition to the presenting disease. Multimodal studies by their nature involved multiple examinations and typically require visits to different healthcare departments. An alternative option is whole body cardiovascular magnetic resonance imaging (WB CVMR), which can assess the heart and arterial network in a single examination, has the advantages of being non-invasive and avoids ionising radiation.

WB CVMR comprises of a suite of cardiac magnetic resonance (CMR) and whole body MR angiography (WB-MRA) sequences, allowing the systemic assessment of whole body atheroma burden, with cardiac structure, function and the detection of myocardial scarring, in a single imaging session. Global atheroma burden has been shown to correlate well with traditional cardiac risk factors, the prevalence of coronary artery disease and future major adverse cardiovascular events [10–13]. Previous studies have assessed the ability of WB-MRA to assess extra-site disease in coronary arterial disease, peripheral arterial disease and vasculitis [14–16]. However a direct comparison of extra-site disease and atheroma burden between cardiovascular disease groups has not been previously conducted. The aim of the study was therefore to determine the yield of WB CVMR in detecting asymptomatic cardiovascular disease, at sites other than at the clinically apparent location in different patient cohorts, and to compare atheroma burden between the groups.

## Methods

### Ethics

The protocols were reviewed and approved by the East of Scotland Research Ethics Committee and was conducted in accordance with the Declaration of Helsinki. All volunteers gave written informed consent to participate in this study.

### Participants

Patients with isolated coronary, cerebrovascular or peripheral vascular disease were identified from existing clinical databases and from local cardiology, stroke, and vascular clinics. Sixty-four participants with a prior diagnosis of single territory vascular disease attended MRI appointments between March 2009 and December 2012. The subjects were categorised based on their history of cardiovascular disease; their demographics are summarised in Table 1. Group 1 contained those with clinical evidence of coronary artery disease (n = 38). Group 2 contained those who had had a cerebrovascular event (n = 9). Group 3 contained those with clinical evidence of peripheral arterial disease (n = 17).

**Table 1:** Demographics and clinical characteristics in the study population

	Group 1	Group 2	Group 3
	CAD	CVD	PAD
Number	38	9	17
Male [%]	31 (81.6)	4 (44.4)	13 (76.5)
Age [years]	66.0 ± 8	61.2 ± 8	68.7 ± 10
Weight [kg]	87.2 ± 13	77.4 ± 12	89.7 ± 28
Height [m]	1.69 ± 0.1	1.64 ± 0.1	1.7 ± 0.1
BMI [kg/m^2^]	30.6 ± 4	28.7 ± 2	28.8 ± 4
Systolic BP [mmHg]	135 ± 15	132 ± 12	137 ± 14
Diastolic BP [mmHg]	76 ± 9	74 ± 8	78 ± 8
Hypertension	32 (84.2)	6 (66.7)	13 (76.5)
Antiplatelet	34 (89)	9 (100)	15 (88)
Antihypertensive	37 (97)	8 (89)	14 (82)
Statin prescription	30 (79)	8 (89)	16 (94)
Type 2 Diabetes	19 (50)	5 (55.6)	6 (35.3)
Non-Smokers	16 (42)	1 (11)	1 (6)
Former-Smokers	20 (53)	8 (89)	11 (65)
Smokers	2 (5)	0 (0)	5 (29)

Values expressed as Mean ± SD, or N (%)

*BMI* body mass index, *BP* blood pressure, *CAD* coronary artery disease, *CVD* cerebrovascular disease, *PAD* peripheral arterial disease

Coronary arterial disease (Group 1) included non-fatal acute myocardial infarction, hospitalised acute coronary syndrome, resuscitated cardiac arrest (not attributed to a non CAD causes), coronary artery bypass graft (CABG) or any other coronary revascularisation procedure. Cerebrovascular disease (Group 2) inclusion criteria were non-fatal strokes or transient ischemic attacks (TIA) confirmed by a specialist stroke physician. Peripheral arterial disease (Group 3) inclusion criteria was ankle-brachial pressure index (ABPI) <0.9 with intermittent claudication, walking distance of not more than 200 yards, or abnormal toe systolic pressure. Volunteers were excluded if they had a clinical history of arterial disease at any site other than their primary diagnosis location. Other exclusion criteria included the possibility of metallic implants, history of claustrophobia, pregnancy, renal replacement therapy, end stage renal disease, therapy for any chronic inflammatory disease, atrial fibrillation or malignancy.

### Magnetic resonance imaging

Images were acquired on a 32 RF receiver channel, 3 Tesla MRI scanner (Magnetom Trio, Siemens, Erlangen, Germany) equipped with high-performance gradient system and electrocardiograph (ECG)-gating. For whole body coverage, a combination of six RF coils were used: head matrix (12 elements); neck matrix (4 elements); spine matrix (up to 24 elements); two body matrix (6 elements each); and peripheral angiography phased array RF surface coils (16 elements. Subjects were placed head first into the magnet bore and were examined in the supine position. Total scan time was of the order of 45 min.

#### Whole body magnetic resonance angiography protocol

For localisation, four low-resolution images were acquired from head to foot using gradient echo fast low angle shot (FLASH) sequences with 500 mm field of view (FOV). Whole body magnetic resonance angiography (WB-MRA) images involved the acquisition of 4 overlapping 3D data sets using a coronal spoiled FLASH (fast low angle shot) sequence (see Table 2 for acquisition parameters). Four anatomically distinct stations with field of view of 500 mm were: head and thorax (station 1), thorax and abdomen (station 2), abdomen and upper legs (stations 3) and lower legs (station 4). These were positioned with an overlap of at least 75 mm between each field of view. Anatomical paired images were acquired pre- and post-contrast. A standard dose of 25 ml gadoterate meglumine contrast agent (Dotarem, Guerbet, Villepinte, France) was administered by intravenous injection in the antecubital fossa in 2 separate boluses (10 ml and 15 ml respectively) using a Spectris Solaris power injector (MedRad, Pittsburgh, USA) at a rate of 1.5 ml/sec, each followed by a 20 ml bolus of saline. For station 1 and 4 imaging, post-contrast images were acquired after an injection of 10 ml of 0.5 mmol/ml gadoterate meglumine contrast agent. Station 1 image acquisition commenced when the bolus of contrast agent arrived at the top of the aortic arch in the ‘2D Care Bolus’ images (Siemens, Erlangen, Germany). Station 4 image acquisition followed immediately after this, with three consecutive volumes of station 4 acquired to ensure that peak arterial enhancement was caught. There was a delay before stations 2 and 3 image acquisition of at least 10 min to allow contrast washout and minimise venous contamination during the second injection and image acquisition [17]. Post-contrast images were acquired after an injection of 15 ml of 0.5 mmol/ml gadoterate meglumine contrast agent, again followed by a 20 ml saline bolus. Acquisition commenced once the contrast agent bolus was seen in the descending aorta in the coronal 2D Care Bolus image. Station 3 imaging commenced immediately after the completion of the acquisition of station 2.

**Table 2:** Imaging parameters for MRI sequences used for the combined CMR and WB-MRA protocol

	Location	Sequence	Plane	TR (ms)	TE (ms)	Flip Angle (^0^)	Pixel size (mm)	Slice thickness (mm)
WB-MRA	Station 1	FLASH	Coronal	2.68	1	19	1.1x1	1.1
WB-MRA	Station 2	FLASH	Coronal	2.6	0.96	16	1.3x1.1	1.3
WB-MRA	Station 3	FLASH	Coronal	3.47	1.21	37	1.5x1	1.4
WB-MRA	Station 4	FLASH	Coronal	2.61	0.96	22	1.2x1.1	1
LVA	Heart LV	TrueFISP	Short axis	3.40	1.48	50-60	1.9x1.4	6
LGE-CMR	Heart LV	PSIR	Short axis	846.4/5.21	1.99	20	1.9x1.4	6

*WB-MRA* whole body magnetic resonance angiography, *LVA* left ventricular analysis, *LGE-CMR* Late gadolinium enhanced cardiac magnetic resonance images, *TR* repetition time, *TE* echo time

#### Cardiac magnetic resonance (CMR) protocols

Cardiac magnetic resonance (CMR) imaging utilised a spine matrix and six-element body array matrix RF coils. Left ventricular assessment involved the acquisition of short axis, multi-slice 2D images from the atrio-ventricular ring to the apex using a CINE TrueFISP sequence with retrospective ECG-gating, with repeated end-expiratory breath-holds (Table 2). The slice thickness was 6 mm and inter-slice gap was 4 mm. Ten minutes after the injection of the first dose of contrast agent, the ECG-gated, breath-hold, end-diastole, short-axis, multi-slice late gadolinium enhanced (LGE) CMR images were acquired using a 2D phase sensitive inversion recovery (PSIR) sequence (Table 2).

#### WB-MRA image analysis

**Fig. 1:**
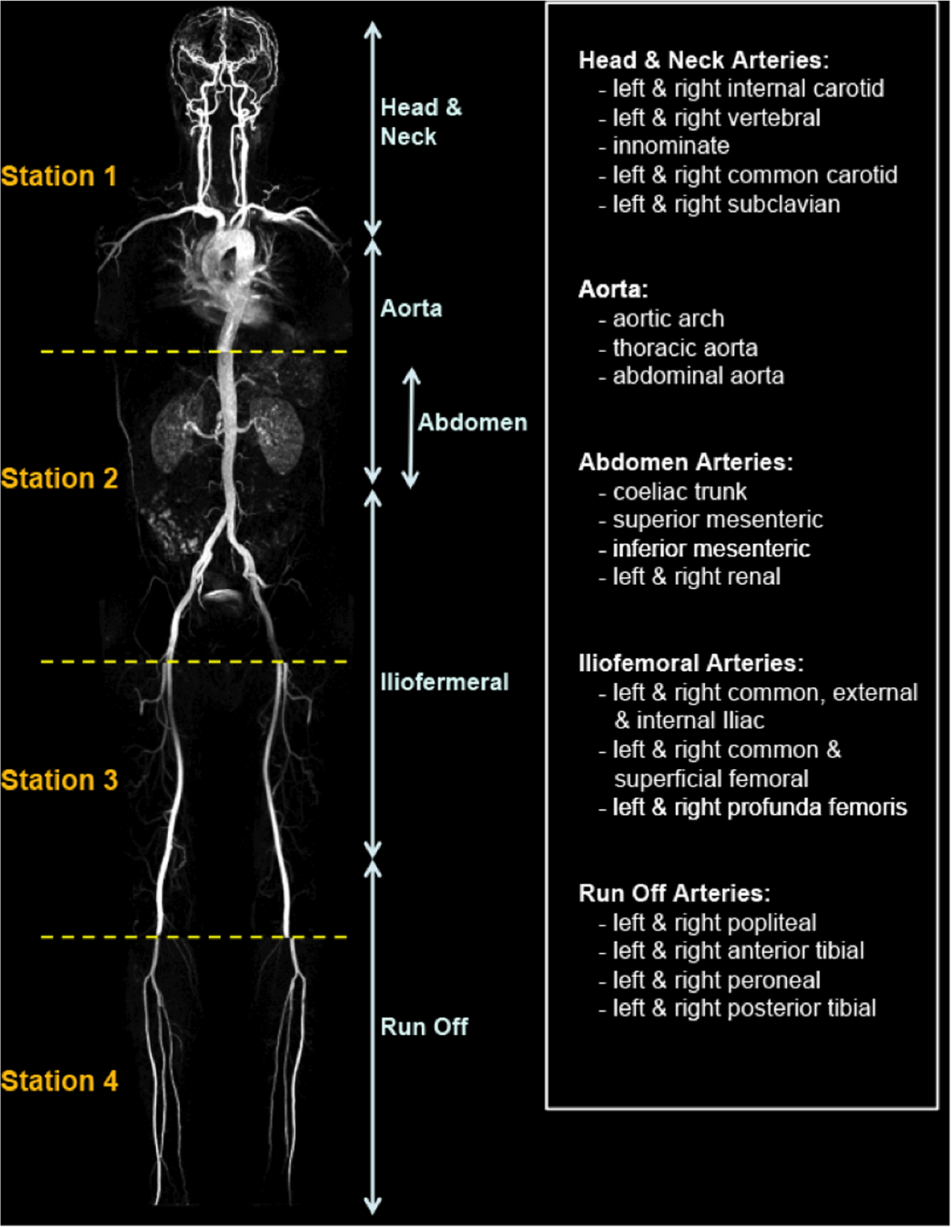
Whole body magnetic resonance angiogram showing the acquisition stations 1 to 4 (left of image) and the 5 arterial territories (right of image). The 31 vessel segments are listed (far right box)

Researchers were blind to the participants’ clinical history during image analysis. The 3D WB-MRA datasets were viewed offline as source images using both multi-planar reconstruction (MPR) and maximum intensity projections (MIP) (Carestream PACS Client Suite Version 10.1 sp1, Rochester, NY, USA) by a radiologist with experience of reporting over 400 whole body magnetic resonance angiograms. Based on previous pilot work, the arterial network was divided into 31 vessel segments extending from the internal and external carotid arteries to the trifurcation vessels of the lower limb (Fig. 1). Each arterial segment was scored according to maximal luminal stenosis within the vessel lumen. Categorical MRA scores from 0-4 were allocated to each vessel segment, where 0 = healthy segment with no stenosis, 1 = <50 % stenosis, 2 = 51-70 % stenosis, 3 = 71-99 % stenosis, 4 = vessel occlusion. For each participant, the ‘standardised atheroma score’ (SAS) was calculated using equation [1] where n is the number of diagnostic segments [18].

**Fig. 2:**
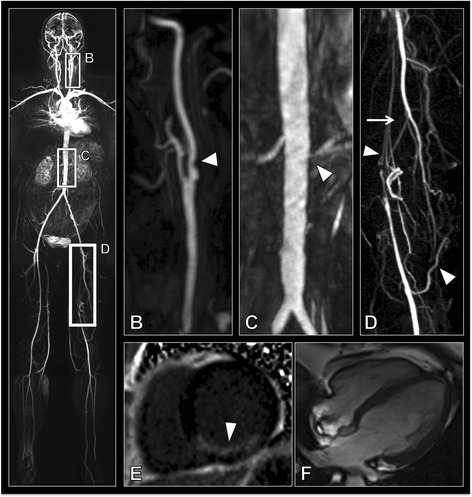
**a** Whole body angiogram of a peripheral arterial disease patient. **b** Sagittal MIP (maximum intensity projection) of the bifurcation of the left carotid artery showing >50 % stenosis of the internal carotid artery (arrow head). **c** Coronal MIP of the renal arteries showing a normal right renal artery but a >70 % stenosis of the left renal artery (arrow head). **d** Coronal MIP showing a long segment occlusion of the left superficial femoral artery (arrow) with extensive collateral formation (arrow heads) with reformation distally. **e** Short axis late gadolinium enhancement showing a large unrecognised myocardial infarct (arrow head). **f** 4 chamber view of the heart showing normal dimensions of the ventricles

1 $$ \mathit{\mathsf{S}}\mathit{\mathsf{A}}\mathit{\mathsf{S}} = \left[\left(\frac{\mathsf{\varSigma}\kern0.5em \mathit{\mathsf{M}}\mathit{\mathsf{R}}\mathit{\mathsf{A}}\ \mathit{\mathsf{scores}}}{\mathit{\mathsf{n}}}\kern0.5em \right)\kern0.75em \div \kern0.5em \mathsf{4}\right]\kern0.75em \times \kern0.75em \mathsf{100} $$

The 31 vessel segments were subdivided into 5 anatomical territories: (i) the head and neck arteries, (ii) the aorta, (iii) the abdominal arteries, (iv) the ilio-femoral arteries and (v) the run off arteries in the lower limbs (Fig. 1). Regional standardised atheroma scores were calculated for each anatomical territory. For each patient the individual vessel segments that had MRA scores of 3 or 4 were noted, with the number of vessels with stenosis greater than 70 % being counted and their location recorded, as luminal narrowing of greater than 70 % is considered clinically significant. In addition a moderate stenosis assessment recorded the number and location of vessel segments that had MRA scores equal to 2 (50-70 % stenosis), in a similar manner to that of the severe stenosis assessment.

#### (ii) CMR image analysis

Left ventricular analysis images were analysed offline using Argus software (Siemens, Erlangen, Germany) by two experienced CMR researchers. The manual 3D digital segmentation involved tracing endocardial and epicardial contours on the short-axis left ventricle images at end-diastolic and end-systolic phases of the cardiac cycle. Papillary muscles were treated as part of the blood pool volume unless they were indistinguishable from the myocardial wall, and then they were assigned as left ventricle muscle. The left ventricular mass (LVM), stroke volume (SV), ejection fraction (EF), end-diastolic (EDV) and end-systolic volumes (ESV) were determined using an algorithm based on the Simpson rule [19]. Results were normalised to body surface area. Left ventricular hypertrophy was defined as an indexed LVM above the normal range for sex [19]. Late gadolinium enhanced images of the left ventricle were inspected for evidence of myocardial signal enhancement using a Carestream PACS workstation (Rochester, NY, USA). The location was recorded according to the AHA 17 segment model [20]. Delayed enhancement was defined as <50 % or >50 % wall thickness according to the maximum depth of delayed enhancement in any segment.

### Statistical methods

Descriptive statistics were used for the analysis of the demographic and clinical features of the cohorts with data expressed as mean ± standard deviation (sd) for normally distributed data, and median (interquartile range) for non-normal distributed data. Normality tests were performed; if the test failed, where possible standard transformations such as square root, reciprocal or logarithmic transforms were used to generate a Gaussian distribution. To test the null hypothesis to determine if samples originated from the same distribution, one-way analysis of variance (ANOVA) with the Bonferroni post hoc adjustment was used for the parametric data, and Kruskal–Wallis ANOVA by ranks was used for the non-parametric data. ANCOVA was performed to confirm differences between the groups with the WB-SAS as the dependant variable. MANCOVA was used to determine the relations of the LV metrics to the demographic data with the LV metrics entered as the dependant variables. Pearson correlation coefficients between WB-SAS, LVA and population demographic metrics were reported. All variables with a p-value <0.3 on univariate analysis were entered into a multivariate regression analysis with the WB-SAS as the dependent variable and the remainder as independent variables. All data were analysed using SPSS statistical package (version 21.0, SPSS Inc. Chicago, Illinois). Significance was assumed when p < 0.05. A local statistician provided statistical support.

## Results

CMR and whole body MRA images were acquired from 64 participants (75 % male, age 66.1 ± 8.5) with single site cardiovascular disease. There were no statistically significant differences in the demographic metrics between each of the diseased groups (Table 1), except for smoking status with significantly more people in the PAD group being current smokers than in either the CAD or cerebrovascular groups.

In the WB-MRA analysis, 1978 of the 1984 vessel segments (99.7 %) were interpretable. 6 segments in 4 of the 64 examinations were rated as ‘non-diagnostic’ due to movement artefact or incomplete vessel visualisation. 619 (31.3 %) of the 1978 arterial segments had evidence of luminal narrowing: 453 (22.9 %) had stenosis below 50 %, 61 (3.1 %) had stenosis between 50-70 %, 63 (3.2 %) had stenosis between 70-90 %, and 42 (2.1 %) had complete occlusion.

The PAD group had the highest whole body standardised atheroma score (WB-SAS) of 24.8 ± 9.9 and the CAD group had the lowest WB-SAS of 7.0 ± 6.2. The WB-SAS of the PAD patients was statistically significantly higher than those of either the CAD or cerebrovascular disease patients (ANOVA test: P ≤ 0.001). Differences between the groups persisted on ANCOVA (F = 15.18, p < 0.001), accounting for age, gender, smoking status, blood pressure, BMI and statin prescription. There was no significant difference in the WB-SAS between the CAD and cerebrovascular disease groups. Across all 5 anatomical territories, the PAD subjects’ regional SAS were consistently higher compared to either the CAD or cerebrovascular groups’ scores (Table 3). There were no statistically significant differences between any of the regional SAS of CAD and cerebrovascular group. On univariate analysis, there were positive correlations of SAS with age (r = 0.37 P = 0.002), smoking status (r = 0.397 P = 0.002), LV ejection fraction (r = -0.26 P = 0.034) and LV mass (r = -0.42 P = 0.001) and a trend towards an association with diastolic blood pressure (r = -0.23 P = 0.052) (Table 4). On multivariate analysis, age (β 0.36 p = 0.002), smoking status (β 0.34 p = 0.002), and LV mass (β -0.61 p = 0.001) continued to demonstrate a significant association with WB-SAS.

**Table 3:** Whole body cardiovascular magnetic resonance imaging (WB CVMR) data for each group including magnetic resonance angiography (MRA) scores, standardised stenosis scores (SAS), left ventricular analysis (LVA) and left ventricular late gadolinium enhancement (LGE) results

	Group 1	Group 2	Group 3
	CAD	CVD	PAD
WB-SAS	4 (2–11.25)	6 (2-10)	24 (17.5-30.5)
Head/Neck-SAS	2.8 (0–5.6)	8.3 (1.4–9.7)	19.4 (9.7-25)
Aorta-SAS	8.3 (0–16.7)	8.3 (8.3-12.5)	8.3 (8.3-16.7)
Abdomen-SAS	8.3 (0–10)	0 (0–5)	20 (5-27.5)
Ilio-Femoral-SAS	6.25 (0–20.8)	4.2 (2.1–14.6)	37.5 (27.1-52.1)
Run Off-SAS	0 (0–10.2)	0 (0–18.7)	31.3 (11-48.5)
LVM (g/m^2^)	59.1 (54.1–64.5)	53.4 (47.4–71.1)	105.7 (67.8-124.5)
EDV (ml/m^2^)	74.7 ± 18.8	72.4 ± 15.2	104.1 ± 34.9
ESV (ml/m^2^)	25.3 (18.3–29.7)	22.4 (17.8–35.8)	33.8 (22.8-42.8)
EF (%)	64.5 ± 9.8	64.7 ± 13.4	66.4 ± 11.8
SV (ml/m^2^)	46.8 (40.4–52)	46 (40.2–52.7)	73.7 (39.4-92.7)
LGE	16 (42.1 %)	0 (0 %)	5 (29.4 %)

Values expressed as Mean ± SD, Median (Interquartile range) or N (%)

*CAD* coronary artery disease, *CVD* cerebrovascular disease, *PAD* peripheral arterial disease, *SAS* standardised atheroma score, *WB* whole body, *LVM* indexed left ventricular mass, EDV indexed end diastolic volume, *ESV* indexed end systolic volume, *EF* ejection fraction, *SV* indexed stroke volume, *LGE* Late gadolinium enhancement

**Table 4:** Correlation of whole body standardised atheroma score with demographic and left ventricular parameters

	Pearson correlation	*P*-value
Age	0.461	<0.001
Gender	-0.19	0.091
Systolic BP	0.19	0.095
Diastolic BP	-0.23	0.052
BMI	-0.22	0.06
Hypertension	0.13	0.18
History of Smoking	0.4	0.002
T2DM	0.05	0.37
Statins	0.008	0.48
Left ventricular metrics		
LV End diastolic volume	0.11	0.22
LV End systolic volume	0.17	0.11
LV stroke volume	-0.18	0.11
Ejection fraction	-0.26	0.03
LV mass	-0.42	0.001

For each patient, the extent of stenosis was further investigated by counting the number of vessel segments that had either (a) severe stenosis with MRA score of 3 or 4 (associated with luminal narrowing of greater than 70 %) or (b) moderate stenosis with MRA score of 2 (associated with luminal narrowing of between 50 and 70 %). The results are summarised in Table 5. 100 % of PAD patients have severe stenosis detectable in at least one arterial vessel, with the ilio-femoral arteries being most affected. A third of the CAD patients had severe stenosis present in their MRA images, with the run off arteries most commonly affected (18.4 % of patients), while severe arterial stenosis was detected in only 22 % of the cerebrovascular patients. With luminal narrowing of greater than 70 % considered clinically significant, this analysis is useful for screening patients and highlighting those who require follow-up investigations.

**Table 5:** The percentage of patients in each group that have at least one vessel segments with (a) severe stenosis (MRA score = 3 or 4) and (b) moderate stenosis (MRA score = 2) for the different anatomical territories. The bracketed number is the total number of vessel segments with the relevant degree of stenosis

Anatomical Territories	(a) Severe stenosis			(b) Moderate stenosis		
	Group 1	Group 2	Group 3	Group 1	Group 2	Group 3
	CAD	CVD	PAD	CAD	CVD	PAD
Whole body (%)	34.2 (26)	22.2 (6)	100 (73)	37 (21)	56 (6)	88 (34)
Head/Neck (%)	5.3 (4)	0 (0)	29.4 (8)	11 (4)	33 (3)	29 (7)
Aorta (%)	0 (0)	0 (0)	5.9 (1)	3 (1)	0 (0)	6 (1)
Abdomen (%)	7.9 (4)	11.1 (1)	52.9 (11)	18 (7)	0 (0)	12 (2)
Ilio-Femoral (%)	10.5 (6)	0 (0)	76 (28)	8 (5)	11 (1)	59 (15)
Run off (%)	18.4 (12)	22.2 (5)	58.8 (25)	8 (4)	22 (2)	41 (9)

*CAD* coronary artery disease, *CVD* cerebrovascular disease, *PAD* peripheral arterial disease

The left ventricular assessment (Table 3) revealed that the PAD group have the largest mean left ventricular mass (LVM), end-diastolic (EDV), end-systolic volume (ESV), cardiac ejection fraction (EF) and stroke volume (SV), and these differences reached statistical significance for the LVM, EDV and SV values (ANOVA P ≤ 0.007). These differences persisted on MANCOVA (F = 8.87, p = 0.001 for LVM, F = 3.27, p = 0.047 for EDV and F = 4.43, p = 0.018 for LVM). The differences between the CAD and cerebrovascular disease cohorts were relatively small and did not reach statistical significance. Left ventricular hypertrophy was detected in 16 % of the study population, while impaired left ventricular systolic function was present in 13 %.

Late gadolinium enhancement (LGE) was observed in 21 (32.8 %) of the 64 subjects. All subjects with LGE had scarring in a subendocardial location with a territorial distribution typical of an ischaemic aetiology. The majority of the enhancement occurred in the CAD group, with 16 (42.1 %) CAD participants displaying evidence of myocardial scarring, affecting a total of 91 AHA segments. No myocardial LGE was observed in the cerebrovascular disease group images. Five (29.4 %) of the PAD group had evidence of unrecognised myocardial infarction (UMI), affecting a total of 21 AHA segments (See Figure 2). UMIs tended to occur in the inferior wall with 9/21 UMIs occurring in the inferior segments, with the second most common location being the inferoseptal segment with 4/21 UMIs occurring in this region. Recognised MIs demonstrated no territorial predominance. UMIs were smaller, involving an average of 4.2 AHA segments, compared with 6.1 AHA segments in the recognised MI group. 40 % of UMIs involved less than 50 % of the myocardial thickness in the affected segments while the remaining 60 % involved greater than 50 % of the myocardial thickness. In comparison, 73 % of recognised MIs involved >50 % of the myocardial thickness. No correlation was observed between WB-SAS and either the presence or the severity of the late gadolinium myocardial enhancement.

## Discussion

We have shown that whole body cardiovascular MRI is a feasible solution to stratify the extent of atherosclerosis in arteries and the extent of cardiac dysfunction and myocardial scarring in a 45-min exam. This is also the first study to show a positive correlation between the whole body atherosclerotic stenotic burden and left ventricular mass.

Stratified medicine has become a topic of increasing importance [21, 22]. This is as important in cardiovascular disease as it is in cancer, and requires accurate definition and quantification of disease. This is especially important given findings that polyvascular disease is associated with significantly higher rates for major adverse cardiovascular events than in single site vascular disease [23]. In our study we have demonstrated the ability of WB-MRA to stratify and quantify atherosclerotic burden throughout the body. Significant undetected arterial stenoses were found to be present in multiple sites in all disease groups. We found a 37 % prevalence of extra-coronary arterial disease in CAD. This is lower than the 50 % reported in one study although this latter study did not exclude patients with known extra-coronary disease [16]. It is also lower than the 55 % reported in a more recent study [12], although this was performed at 1.5 T with a lower spatial resolution than obtained in the current study, thus the improved arterial definition may have led to a more accurate quantification of the degree of stenosis due to improved spatial resolution found at 3 T. We found that 33 % of cerebrovascular disease patients had significant disease outwith the head and neck. This is significantly lower than that reported by Paraskevas et al. [5], however their study only looked at patients with known unilateral internal carotid artery occlusion which is at the extreme end of the carotid disease spectrum, while our study used prior cerebrovascular events as inclusion criteria. Our PAD cohort demonstrated significant disease above the level of the abdominal aortic bifurcation in 47 % which is slightly higher than that previously reported on WB-MRA [15].

The standardised atheroma score was significantly higher in the peripheral arterial disease group than either the cerebrovascular or the coronary arterial disease group. Previous epidemiological studies have shown that patients with PAD have a 33 % higher composite risk of CV death, myocardial infarction, stroke, or hospitalisation for atherothrombotic event(s) than either cerebrovascular or coronary arterial disease groups [24]. As well as the intrinsic risk of arterial stenosis, part of the causative mechanism may be the effect the atherosclerotic burden has on the heart. We have shown for the first time the association between whole body arterial atheroma burden and left ventricular mass, which is known to be strongly associated with future cardiovascular events [25]. This may be due to the stiffening nature of the atherosclerosis on the arteries, as total atheroma burden has been shown to correlate with arterial stiffness, [26] which in turn is associated with left ventricular hypertrophy [27]. Given that both left ventricular mass and atheroma burden are associated with increased future risk of cardiac events, further work is required to extricate the interaction between these measures, to ascertain whether these need to be targeted individually, or whether there is a single linking aetiology which can be targeted.

Unrecognised myocardial infarctions are present in 29.4 % of patients with peripheral arterial disease. The rate in peripheral arterial disease patients is significantly higher than the 6 % reported in a previous PAD population using ECG and echocardiography during pre-operative work-up [28], or the 14 % reported in a series of patients undergoing pre-operative coronary angiography screening [29]. However ECG has been shown to only detect 6-29 % of unrecognised myocardial infarcts revealed on late gadolinium enhancement [30, 31]. Our observed incidence is closer to that expected from a previous study looking at whole body cardiovascular MR in a population cohort study which showed rates of unrecognised myocardial infarction in 19.7 % of 70 year olds and 30 % in 75 year olds [30, 32]. That the prevalence in the PAD population was on par with a 75 year old cohort despite having a mean age of 68 is in keeping with the higher prevalence of risks factors in our population, although a recent study has called into question the link between unrecognised myocardial infarctions and traditional risk factors [33]. None of the patients with cerebrovascular disease had evidence of unrecognised myocardial infarcts. This is surprising given the results of previous studies showing unrecognised myocardial infarcts in 32 %-52 % of patients but may be due to the small numbers of this group in the current study.

Recognition of unrecognised myocardial infarction is important as these have the same prognostic implications as recognised myocardial infarcts [34]. Furthermore these patients respond well to both conventional secondary prevention medication and percutaneous coronary intervention [35–37].

It could be argued that since patients with clinically apparent cardiovascular disease in one site will result in patients being treated for atherosclerotic risk factors that further information about disease elsewhere is superfluous. However this ignores several factors. Despite our study showing comparable rates of statin prescriptions between the different groups, a previous population study has shown that community prescription of risk modifying agents is markedly different between disease groups, with fewer patients with PAD being prescribed statins and antiplatelet agents compared with stroke or coronary artery disease groups [38]. This suggests poor appreciation of the extensive disease present elsewhere in the body, indeed, in our study the PAD population had the most extensive extra-primary site disease. The second is the additional prognostic information this provides. Multisite disease is associated with a significantly raised risk of future major adverse cardiac and cerebrovascular events (MACCE) compared with single site disease, and had a greater detrimental effect on future prognosis than the presence of diabetes [23, 38]. While these studies have focused on symptomatic disease, the significance of asymptomatic disease is supported by recent studies showing increased risk of MACCE in patients with higher global atherosclerotic burden on whole body MR angiography [10, 11]. Thus patients with polyvascular disease may warrant more intensive management and follow-up as well as being ideal candidates for future novel therapeutic agents [24]. For those being referred for a clinical MRA of a specific vascular territory, extension of this to a whole body cardiovascular MR would be a logical step, and indeed the added cost of extending a clinically indicated MRA to include the rest of the body is small when considered in relation to the high cost of the baseline exam, and in a peripheral arterial disease population has been shown to be cost effective due to its reductions in requirements for other imaging investigations (such as echocardiography and carotid Doppler) and alterations in patient management, although other cardiovascular disease cohorts have still to be assessed [39].

The limitations of the current study are: The study groups are unequal in size with a relatively small number of cerebrovascular patients and large number of coronary arterial disease. In addition the cerebrovascular group demonstrates a disproportionate number of females which could bias the results, however we demonstrated no significant correlation between SAS and sex, and accounting for sex using an analysis of covariance did not change the results. An intrinsic limitation of WB-MRA is its ability to only provide information on the prevalence of stenotic atherosclerosis, and will thus miss the earliest stages of the disease including vessel stiffening and remodelling. Additionally, while the prognostic effect of symptomatic multisite atherosclerosis is known, the effect of asymptomatic multisite disease still requires further work to elucidate.

## Conclusion

WB CVMR is an effective method for the stratification of cardiovascular disease. The high prevalence of asymptomatic arterial disease, and silent myocardial infarctions, particularly in the peripheral arterial disease group, demonstrates the importance of a systematic approach to the assessment of cardiovascular disease.

## Abbreviations

AHA: American Heart Association
CAD: coronary artery disease
CMR: cardiac magnetic resonance imaging
CVD: cerebrovascular disease
EDV: end diastolic volume
ESV: end systolic volume
LGE-CMR: late gadolinium enhanced cardiac magnetic resonance images
LVA: left ventricular analysis
LVH: left ventricular hypertrophy
LVM: left ventricular mass
MRA: magnetic resonance angiography
PAD: peripheral arterial disease
SAS: standardised atheroma score
SV: stroke volume
UMI: unrecognised myocardial infarct
WB-CVMR: whole body magnetic cardiovascular magnetic resonance
WB-MRA: whole body magnetic resonance angiography
